# A three-step framework for capacity planning in a nursing home context

**DOI:** 10.1080/20476965.2022.2062461

**Published:** 2022-05-21

**Authors:** Nanne A. Dieleman, Martijn Buitink, René Bekker, Dennis Moeke

**Affiliations:** a Operations Analytics, Vrije Universiteit Amsterdam, Amsterdam, The Netherlands; b Statistics Department, de Nederlandsche Bank, Amsterdam, The Netherlands; c Department of Mathematics, Vrije Universiteit Amsterdam, Amsterdam, The Netherlands; d Research Group Logistics & Alliances HAN University of Applied Sciences, Arnhem, The Netherlands

**Keywords:** Nursing homes, capacity planning framework, client-centred care, mixed integer linear programming, genetic algorithm

## Abstract

This paper presents a three-step conceptual framework that can be used to structure the care-related capacity planning process in a nursing home context. The proposed framework provides a sound practical vehicle to organise client-centred care without overstretching available capacity. Within this framework, an MILP for shift scheduling and a Genetic Algorithm (GA) for task-scheduling are proposed. To investigate the performance of the proposed framework, it is benchmarked against the current situation. The results show that considerable improvements can be achieved in terms of efficiency and waiting time. More specifically, it is shown that very modest waiting times can be achieved without exceeding available capacity, despite the fluctuations in care demand across the day.

## 1. Introduction

The Dutch population is ageing and according to the forecasts this trend is likely to continue for the foreseeable future. It is expected that the percentage of people aged 65 years and over will rise from 19% in 2018 to 29% in 2040. In addition, the total Dutch population aged 80 years and over is expected to reach 10% by 2040, compared with 5% in 2018 (PopulationPyramid, 2020; Statistica, 2018). The ageing of the Dutch population puts pressure on the financial sustainability of the Dutch long-term care (LTC) system for two main reasons. Firstly, research demonstrates that the risk of poor health and related physical or mental disability increases with age (Meerding et al., 1998). On average, elderly people over the age of 85 consume four times as much healthcare per person as those aged between 65 and 75 (NZA, 2018). The second reason is that the relative increase in the number of elderly will lead to a substantial increase of the old-age dependency ratio, which measures the number of elderly people at an age when they are generally economically inactive, as share of those of working age.

On top of an increase in demand, the Dutch long-term care labour market is in shortage, and it is expected that the current shortage will increase from over 30,000 in 2015 to more than 90,000 employees in 2030 (Actiz, 2021). Consequently, in order to ensure the long-term financial sustainability of the Dutch healthcare system, providers of long-term care are challenged to develop (innovative) strategies and approaches that enable them to meet the (future) needs of their clients in a more efficient manner. In the Netherlands, nursing homes play an important role in the long-term care continuum. A nursing home can be described as a facility with a domestic styled environment that provides 24-hour functional support and care for persons who require assistance with activities of daily living and who often have complex health needs and increased vulnerability (Sanford et al., 2015, p. 183). Most nursing home clients are in need of assistance with basic activities of daily living due to physical or psychological disabilities. In everyday practice this means that, in order to live their lives according to their own daily routines, nursing home clients greatly depend on timely delivery of care and support. As such, the coordination and timing of service delivery has a significant impact on their perceived quality of life (Moeke, 2016).

Capacity planning plays a vital role in the pursuit of balancing the timely delivery of the required care with the cost of providing that care. When it comes to capacity planning in a nursing home context, the effective and efficient utilisation of the available care workers plays a dominant role. This is due to the fact that care workers are responsible for the daily care and supervision of the residents and their labour costs account for a significant proportion of the total healthcare expenditure (Di Giorgio et al., 2014). Hence, the main focus of capacity planning in a nursing home setting is on getting the right number of care workers with the right set of skills on the right job at the right time.

In addition, Dutch nursing homes are, slowly but surely, becoming information-intensive enterprises. Due to the increasing use of electronic health records and other forms of health information technology, nursing homes have access to large amounts of clinical and operational data. This development enhances data-driven decision-making and process improvement. Also, when it comes to capacity planning, nursing homes are starting to recognise data-driven decision-making as essential to improve their (future) operations (Moeke & Bekker, 2020). Still, in the current nursing home practice, decisions regarding the allocation of care workers are often addressed without a sound quantitative basis (Bekker et al., 2019; Moeke et al., 2016).

### 1.1. Contribution and outline

The main contribution of this paper is that we apply and substantiate a conceptual framework that can be used to structure the care-related capacity planning process in a nursing home context. We do this with the help of empirical data from a real-life case. Using benchmarks that are based on the current situation, we illustrate that substantial improvement can be achieved in terms of efficiency and waiting time. Within the conceptual framework, we envisage the modifications required to existing methods as a secondary, though smaller, contribution. In particular, we present an MILP for shift scheduling, which can be used in a setting with hierarchical qualifications. Furthermore, we develop a Genetic Algorithm (GA) for task scheduling based on the time preferences of the clients and the availability of care workers. The combination of these features is uncommon in the scheduling literature. Also, in our GA, the starting times of tasks (and thus the fitness) are determined using an LP. This differs from traditional GAs.

We note that our focus is on care by appointment. These are care tasks for which, based on the needs and preferences of the client, it is possible to make a fairly detailed planning in advance (Moeke, 2016). Examples are giving medicine and help with getting out of bed in the morning.

The remainder of this paper is structured as follows. Section 2 presents related literature. In Section 3, the context related to the real-life case under study is being described. Next, in Section 4, our three-step framework is presented, where Sections 4.1, 4.2 and 4.3 elaborate on the modelling aspects in the different steps of the three-step framework. More specifically, an MILP for shift scheduling and a GA for task-scheduling are proposed in Sections 4.2 and 4.3, respectively. Section 5 presents the numerical results using the real-life case data. In the last section, conclusions are drawn, and implications for practice and future research directions are discussed.

## 2. Related literature

There are numerous Operations Research (OR) studies on capacity planning in healthcare. However, to date, the area of nursing home care has received hardly any attention. As such, the findings of Hulshof et al. (2012) still hold. They state that “The body of OR/MS literature directed to residential care services is limited”.

### 2.1. Shift scheduling

In most service systems, demand fluctuates during the course of the day. In order to develop an appropriate shift schedule, first the expected workload needs to be predicted and translated into the required number of employees during the course of a day such that the desired service level is met. Shift scheduling is concerned with apportioning the required staffing levels into shifts that are specified by their start times, lengths, the number, and type of employees, and timing of (lunch) breaks. A shift schedule should adhere to capacity restrictions, service-level requirements, and (working hours) regulations.

Shift scheduling methods have been applied to transportation systems (De Bruecker et al., 2018; Ciancio et al., 2018; Solos et al., 2016), emergency services (Becker et al., 2019; Butler & Maydell, 1979; Rajagopalan et al., 2011), call centres (Bhulai et al., 2008; Koole & Van Der Sluis, 2003), healthcare systems (Brunner et al., 2009; Omar et al., 2015; Siferd & Benton, 1994) and many other types of service organisations. See also, Erhard et al. (2018) for a rather recent review on physician scheduling. However, the work of (Bekker et al., 2019) is the only study we found that examined shift scheduling in a nursing home setting. In their study, they developed a Mixed-Integer Linear Programming (MILP) model using a Lindley-type equation and techniques from stochastic optimisation. The results of their numerical experiments show substantial improvements both in terms of average waiting time as well as in service level. In addition, the proposed shift schedules resulted in a more evenly spread workload for the care workers.

For the purpose of this study, we extend the approach of Bekker et al. (2019) by including differentiated practice. Moreover, we incorporate the assignment of care tasks to shifts (i.e., task assignment).

### 2.2. Task assignment

Task assignment can be regarded as a subproblem of workforce planning, dealing with “combining individual tasks into task sequences that could usefully be carried out by one person” (Ernst et al., 2004, p. 5). In this study, the focus of task assignment lies with assigning care tasks to specific shifts (and thus care workers).

The assignment of tasks to shifts shows similarities with the Unrelated Parallel Machine Scheduling Problem (UPMSP), for which there is a considerable amount of the literature available, see, e.g., Allahverdi et al. (2008). Most studies on machine scheduling focus on minimising the makespan, whereas in this contribution we focus on delivering care as close as possible to the time preference of the nursing home resident (i.e., minimising earliness and lateness). This may be interpreted as parallel machine scheduling with different due dates. We refer to Ark et al. (2022) for a recent study in the case of common due dates; their literature review hardly shows references to studies in which tardiness costs are minimised in the case of different due dates. Another complicating factor in the setting under study is that care workers only work part of the day, leading to the phenomenon of unavailability of “machines”. Finally, we note that the list of solution methods in (Allahverdi et al., 2008,) shows that metaheuristics are often used, including genetic algorithms.

**Table 1.** Qualification levels.

Qualification	Tasks to be carried out
level	
QL1	Bringing food and drinks, cleaning, transferring, bed cleaning
QL2	Getting in/out of bed, eating, toileting, making the beds, washing
QL3	Giving medication, simple medical check ups

**Table 2.** Duration of care tasks.

	Care durations (in minutes)	
	Average	St dev.
Total	14.38	7.16
QL2	16.42	6.83
QL3	12.31	6.83

Some prominent task assignment studies involving time windows are Gertsbakh & Stern (1978), Mankowska et al. (2014), and Gertsbakh & Stern (1978) discuss task scheduling with time windows for a homogeneous workforce. The difference is that they do not incorporate penalties for delay. Their objective is to find the minimum required staffing requirements to obtain a feasible schedule. Mankowska et al. (2014) consider a home care setting where care workers visit clients at home and time windows reflect the time preferences of their clients. The objective is to minimise the weighted sum of total travel times, total tardiness, and maximum tardiness. Their solution is based on an ILP for small instances and a heuristic for larger ones.

An elementary difference with home care is that travel times are much less important in a nursing home setting. Consequently, travel times are not part of our objective function, whereas they are crucial in home care. Moreover, compared to home care, the workload across the day can be predetermined, which allows us to decompose the capacity planning process into multiple steps, that is, our three-step framework. Such an approach is not (directly) applicable in a home care setting, as the workload depends on the route. We refer to Fikar & Hirsch (2017) for a rather recent overview on home healthcare to Di Mascolo et al. (2021) for a bibliometric analysis, containing many references. Also, in this domain, metaheuristics is the common solution method, see e.g. Fikar & Hirsch, 2017, .

Another distinguishing feature of task scheduling in nursing homes is that qualification levels should be taken into consideration (Bellenguez-Morineau & Néron, 2007; Krishnamoorthy et al., 2012; Schimmelpfeng et al., 2012). However, Bellenguez-Morineau & Néron (2007) focus on the makespan as objective, whereas Krishnamoorthy et al. (2012) consider the minimum workforce for a feasible schedule in case of hard constraints for start times. Finally, Schimmelpfeng et al. (2012) present a task scheduling approach for rehabilitation hospitals with different qualifications and precedence constraints between tasks, but they do not consider time preferences for individual tasks.

The problem studied in Lieder et al. (2015) is most closely related to the problem at hand. They also focus on the assignment of tasks, with different levels of qualification, in a nursing home setting. To optimally solve this scheduling problem, they propose a Mixed Integer Program (MIP) and a Dynamic Programming (DP) approach. However, the state space of the presented DP approach suffers from the curse of dimensionality. As such, the computation time of this approach may become prohibitively long.

### 2.3. Genetic algorithms

Task assignment, as discussed in Subsection 2.2, typically involves NP-hard problems. As a result, there is a vast amount of the literature considering metaheuristics for solving realistic-sized instances. Genetic algorithms are randomised optimisation algorithms, belonging to the class of metaheuristics. One of their primary properties is that they have the ability to maintain a diverse set of solutions to escape from local optima (Eiben & Smith, 2015, Section 3.7). Due to their versatility and adaptability, they can be applied to solve problems in different fields, such as health care (De Carvalho Filho et al., 2014), manufacturing (Gen & Lin, 2014), design (Hornby et al., 2011) and finance (Mahfoud & Mani, 2000).

GAs are highly suitable to solve scheduling and task assignment problems, which is supported by a rich literature. For example, Sakawa & Mori (1999) use an GA to solve job-shop scheduling problems in which processing times and due dates are fuzzy. In Wang et al. (2017), an GA is used to solve dynamic scheduling problems in which two types of costs have to be optimised, which thus results in a multi-objective problem.

GAs have also been used to create nurse schedules. For example, Jan et al. (2000) use a genetic algorithm to create monthly nurse schedules. They consider a range of different (hard and soft) constraints, such as nurses’ preferences and the right of having days off. However, they do not consider qualification levels. The constraint closest to this is that the professional level of the nurse is taken into account, but this is only a soft constraint. In Aickelin & Dowsland (2004) a weekly schedule is created for hospital wards up to 30 nurses. They use an GA that solves the unconstrained version of the problem, and then a decoder that creates the feasible schedules. This is different from our approach in which feasible schedules are obtained by the GA and the LP fitness calculation. The combination of GA and mathematical programming is sometimes also referred to as matheuristic. In Amindoust et al. (2021) a genetic algorithm is created that also incorporates a fatigue factor due to the Covid-19 pandemic. They assume that all nurses have identical skills and develop weekly and monthly schedules.

It should be noted that these papers focus on nurses working in hospitals, which is different from nurses working in a nursing home (in terms of activities, duration of activities, spread of activities over the day, etc.). Moreover, most papers focus on weekly and monthly schedules of the nurses, instead of the daily activity schedules that we consider.

### 2.4. Framework and conclusions

As there are many planning and control decisions in complex (health care) organisations, various frameworks for operations management decisions have been proposed, see, e.g., Hans et al. (2012), Matta et al. (2014), and Vissers et al. (2001). Such frameworks reveal the need to decompose the complex planning process into manageable proportions. The focus of this study is on establishing a blueprint for the shift schedule(s), as well as an operational planning concerning routes for care workers. With the framework of Moeke & Bekker (2020) as starting point, we provide specific methods for each step and verify its value using real-life data.

To summarise this literature section, it can be stated that the literature on planning of nursing home capacity is scarce. Moreover, decisions related to nursing home capacity are involved, as shift and task scheduling are intertwined. Both shift and task scheduling have been addressed in the literature, but the nursing home setting has specific features leading to different problems. For instance, for shift scheduling, there are no papers that address hierarchical shift scheduling in a nursing home context. Moreover, although metaheuristics are common for task assignment problems, the application of an GA in which residents have time preferences and care workers are partly available is not. Finally, incorporating an ILP within the GA differs from traditional GAs.

## 3. Case description

In this study, the emphasis lies on the capacity planning of a Dutch nursing home department during daytime (7:00–23:00). The concerned department provides 24/7 care and support to 18 clients who are all aged 70 years and over. Although all residents need some assistance with activities of daily living, due to somatic and/or psycho-geriatric illnesses, most of them are still largely self-sufficient. The available amount of capacity, in terms of care hours, largely depends on the so-called Care Intensity Package score (in Dutch “het zorgprofiel”) of the clients. For the clients of this department, there is a budget for 2 hours of care and support per resident during daytime, yielding a total available budget of 36 care hours during daytime per day.

The available care workers are hierarchically divided into distinct qualification levels (QLs). This so-called differentiated practice is based on a distinction in education, responsibility, and complexity of care (Jansen et al., 1997). Table 1 shows the QLs that are relevant for this study and the corresponding tasks. The preferred lengths of shifts during which care workers carry out activities are 4, 6, and 8 hours. In order to make it possible for the clients to live their lives according to their preferences, the aim is to deliver the necessary care as close as possible to their time preferences, i.e., minimising delay. The required care (activities) and corresponding time preferences are inventoried on a regular basis, using a standardised, systematic method. During daytime, there are slightly over 100 care activities per day. For more details about the current situation, we refer to Section 5.

## 4. Conceptual framework: a three-step approach

**Figure 1.**
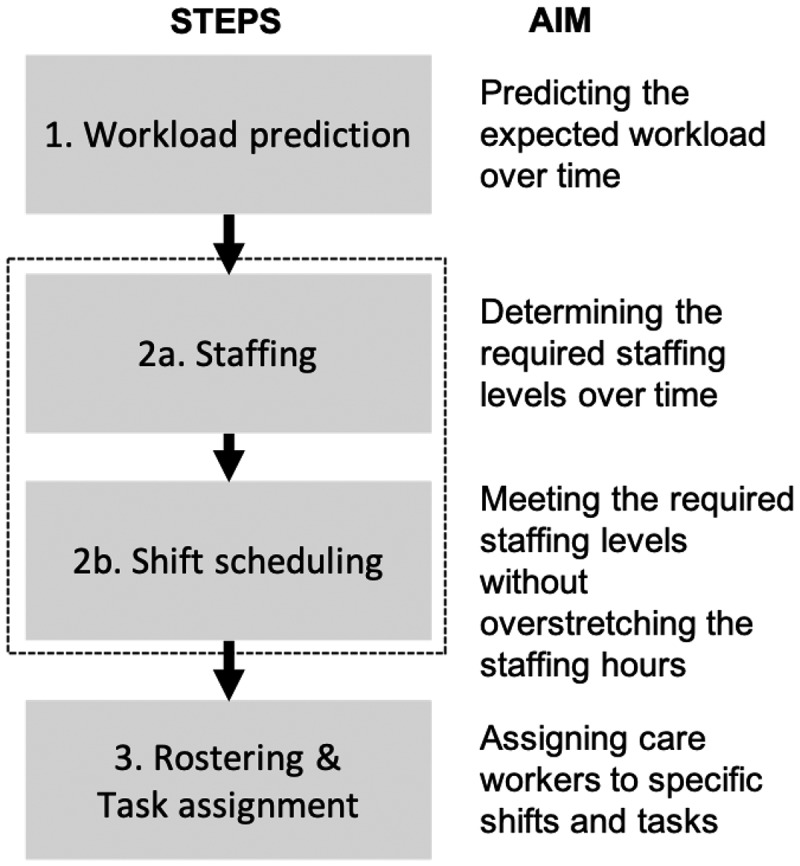
Stages of the care-related capacity planning process.

Based on the recent work of Moeke & Bekker (2020), we apply a three-step framework for capacity planning in a nursing home context (see, Figure 1). More specifically, we identify the following steps: (1) workload evaluation, (2a) staffing, (2b) shift scheduling, and (3) rostering & tasks assignment.

In the first stage (i.e., workload evaluation) the aim is, with the help of historical demand data, to determine the workload over time. This may seem obvious, but practice shows that insight into health care demand is often lacking in long-term care settings. During daytime, the majority of the demand is related to activities of daily living (ADLs). Although there may be strong fluctuations in ADL-related demand, it is possible to collect the time preferences of each client regarding ADLs and estimate the corresponding care durations. Combining the time preference of an activity with the duration provides an estimate of the workload, i.e., the number of care workers required to meet the care demand.

The workload evaluations form the basis for steps 2a and 2b (i.e., staffing and shift scheduling). When it comes to staffing (step 2a) the focus lies on determining the corresponding staffing levels over time in order to meet the demand. The aim of shift scheduling (step 2b) is to determine working shifts (start and end times, breaks, etc.), together with the assignment of the number and type of care workers to each shift, without over-stretching the available staffing hours. In the nursing home setting, we combine staffing and shift scheduling, as the workload can be estimated pretty well during the day time. In other application domains, for example, in call centres, determining staffing levels (step 2a) is done separately due to complexity as a result of uncertainty in demand.

Finally, in step three, the focus lies on assigning care workers to specific shifts and tasks. More specifically, it deals with the following two questions: Which of the available care workers should be assigned to which shift(s)? And, which care tasks should be assigned to which shift(s) in order to meet the demand of the nursing home clients as closely as possible? Assigning care workers to shifts, i.e., rostering, is a classical component in workforce management, see, e.g., Burke et al. (2004); therefore, we focus on the assignment of tasks to shifts (and thus to care workers) in this paper. In the sequel, we elaborate on steps 1, 2, and 3 in Sections 4.1, 4.2, and 4.3, respectively.

### 4.1. Step 1: workload evaluation

This section concerns step 1 of the conceptual framework visualised in Figure 1. The key initial step in capacity planning is to obtain insight into the demand (or workload). The dataset that is used to analyse the workload in the current situation consists of the following variables:

• Resident ID – the ID of a specific resident.

• Preferred Activity Time (PAT) – the preferred starting time of the healthcare activity.

• Task description – a brief description of the activity (i.e. healthcare task) entered as free text.

• Qualification Level (QL) – the QL required to perform the task.

• Expected service time – expected duration of the activity in minutes.

Let N denote the number of activities. We now define notation of the information that needs to be collected from all clients: (i) $T_{n}$ the PAT of activity n, (ii) $QL_{n}$ the required QL of activity n, and (iii) $B_{n}$ the duration of activity n, for n=1,…,N. Based on the input, the first step is to determine the workload $L_{it}$, where $L_{it}$ is the number of activities of QL i at time t if each activity would start at their PAT. In particular,

$$L_{it}=\sum_{n:\;T_{n}\leq t}1(T_{n}+B_{n}>t;\;QL_{n}=i)$$

with 1(⋅) the indicator function. That is, the workload at time t is the number of residents who need care at time t ignoring capacity constraints. As such, it prescribes the required number of care workers at any time if demand would have been met directly (no waiting is allowed). In the current situation, there are 105 tasks, of which 53 are QL2-tasks and 52 are QL3-tasks.

**Figure 2.**
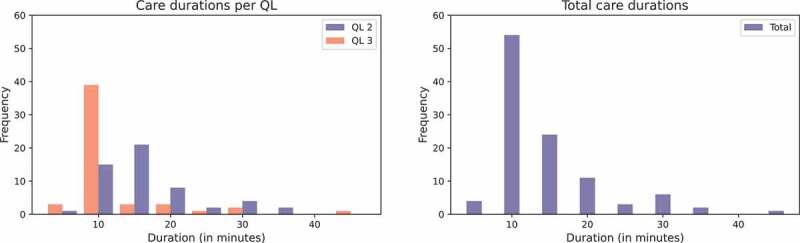
Durations of activities; QL2 and QL3 separate (left) and combined (right).

When it comes to the duration of care tasks, in the current situation, the total average (expected) duration for QL2 and QL3-tasks are 16.42 and 12.31 minutes, respectively, with a standard deviation of 6.83 minutes, which are almost the same for both QLs (see also Table 2). As can be observed in Figure 2 most care tasks take between 10 and 15 minutes. Nevertheless, there is some variation in the duration with tasks that may take well over half an hour.

**Figure 3.**
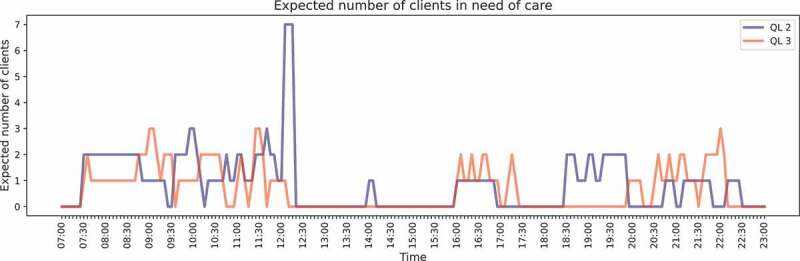
Workload evaluation QL2 and QL3 for the current situation (step 1).

The workload (i.e., the number of residents in need of care) for the current situation is visualised in Figure 3. It can be observed that the workload is relatively high between 7:30 and 12:30, 18:30 and 23:00. Due to the (predictable) fluctuations in workload across the day, capacity planning is non-trivial and requires a solid quantitative foundation.

### 4.2. Step 2: shift scheduling with hierarchical qualifications

In this section, we present the shift scheduling model formulation in case of hierarchical qualification levels; that is, we consider step 2 of the framework presented in Figure 1. The model is applied to a specific nursing home department, but the model can be easily customised by modifying and adding constraints. The model is largely inspired by the MILP formulation in Bekker et al. (2019), but the formulation is extended to allow for different (hierarchical) qualification levels. More specifically, in the shift scheduling phase, we focus on the workload dynamics at an aggregate level. That is, we do not yet distinguish unique care workers or residents, but only consider the total demand and capacity during a time interval. We discretise time and evaluate the aggregate dynamics between epochs t−1 and t, for t=1,2,…,T. In our case study, we choose to consider intervals with a length of 5 minutes.

Note that the total demand follows directly from the workload evaluation $L_{it}$ in step 1. Therefore, let us now first focus on the capacity for a given combination of shifts. For each QL, there are a limited number of shift types. Here, a shift type refers to the combination of starting time of a shift and the length of the corresponding shift (and may also include breaks). Let $K_{i}$ be the total number of shift types for QL i, and let $x_{ik}$ be the number of care workers with QL i that are scheduled for shift type k. We denote by $a_{itk}=1$ that shift type k for QL i works during interval t, and let $a_{itk}=0$ otherwise. Hence, via $a_{itk}$ the user specifies how shift type k for QL i looks like. Then, the available capacity of QL i during interval t is $c_{it}=\sum_{k=1}^{K_{i}}x_{ik}a_{itk}$, for t=1,2,…,T.

At an aggregate level, the difference between the demand and capacity provides the backlog in the amount of work. Let $q_{it}$ denote the backlog of activities requiring QL i at the start of interval t. A complication is that care workers of QL i may perform activities requiring QL 1,…,i. As such, let $c_{it}^{*}$ denote the total number of care workers that perform activities of QL i during interval t. Observe that $c_{it}^{*}$ and $c_{it}$ are not necessarily equal. Now, the aggregate backlog for QL i satisfies the recursive relation $q_{i,t+1}=\max\{0,q_{it}+L_{it}-c_{it}^{*}\}$. Observe that the relation between $q_{i,t+1}$, and $q_{it}$, $L_{it}$, and $c_{it}^{*}$ is linear up to the max{0,⋅} operator. Incorporating such a max{0,⋅} operator in an MILP is quite standard.

We now specify the MILP for determining a shift scheduling (step 2); the notation is given in Table 3.

(1) $$Minimise\;\;\;\sum_{i\in I}\sum_{t\in T}q_{it}$$

(2) $$subject\;\;to\;\;\;c_{it}=\sum_{k=1}^{K_{i}}x_{ik}a_{itk},\;\;\;\;\;\;\;\;\;\;\;\;\;\;\forall i\in I,t\in T$$

(3) $$\sum_{t=1}^{T}c_{it}\leq {\overset{ˉ}{C}}_{i},\;\;\;\;\;\;\;\;\;\;\;\;\;\;\;\;\;\;\;\;\;\;\;\;\;\;\;\;\;\;\;\;\;\;\;\;\;\;\;\;\;\;\;\;\;\;\;\;\;\;\forall i\in I$$

(4) $$c_{it}^{*}\leq \sum_{i{\;}^{\prime }\in I:i{\;}^{\prime }\geq i}c_{i{\;}^{\prime },t},\;\;\;\;\;\;\;\;\;\;\;\;\;\;\;\;\;\;\;\;\;\;\;\;\;\;\;\;\;\;\;\;\;\;\;\;\;\;\;\;\;\;\;\;\forall i\in I,t\in T$$

(5) $$\sum_{i\in I:i\geq i_{min}}c_{it}^{*}\leq \sum_{i\in I:i\geq i_{min}}c_{it},\;\;\;\;\;\;\;\;\;\;\;\;\;\;\;\;\;\;\;\;\;\;\;\;\;\;\;\;\forall i_{min}\in I,t\in T$$

(6) $$q_{i,t+1}\geq q_{i,t}+L_{it}-c_{it}^{*},\;\;\;\;\;\;\;\;\;\;\;\;\;\;\;\;\;\;\;\;\;\;\;\;\;\;\;\;\;\forall i\in I,t\in T$$

(7) $$q_{i,T+1}=0,\;\;\;\;\;\;\;\;\;\;\;\;\;\;\;\;\;\;\;\;\;\;\;\;\;\;\;\;\;\;\;\;\;\;\;\;\;\;\;\;\;\;\;\;\;\;\;\;\;\forall i\in I$$

(8) $$c_{it}\geq C_{i}^{\min},\;\;\;\;\;\;\;\;\;\;\;\;\;\;\;\;\;\;\;\;\;\;\;\;\;\;\;\;\;\;\;\;\;\;\;\;\;\;\;\;\;\;\;\;\;\;\;\;\forall i\in I,t\in T$$

(9) $$q_{it}\geq 0,\;\;\;\;\;\;\;\;\;\;\;\;\;\;\;\;\;\;\;\;\;\;\;\;\;\;\;\;\;\;\;\;\;\;\;\;\;\;\;\;\;\;\;\;\;\;\;\;\;\;\;\;\;\;\;\forall i\in I,t\in T$$

(10) $$x_{ik}\in N_{0},\;\;\;\;\;\;\;\;\;\;\;\;\;\;\;\;\;\;\;\;\;\;\;\;\;\;\;\;\;\;\;\;\;\;\;\;\;\;\;\;\;\;\;\;\;\;\;\;\;\;\;\;\forall i\in I,k\in K$$

(11) $$c_{it},c_{it}^{*}\in N_{0},\;\;\;\;\;\;\;\;\;\;\;\;\;\;\;\;\;\;\;\;\;\;\;\;\;\;\;\;\;\;\;\;\;\;\;\;\;\;\;\;\;\;\;\;\;\;\;\forall i\in I,t\in T$$

**Table 3.** Notation for shift scheduling problem.

Notation for shift scheduling problem	
Sets	
T	Number of time epochs
I	Number of qualification levels
$K_{i}$	Total number of shift types of care workers with QL i
Parameters	
$a_{itk}$	1 if shift type k with QL i works interval t, 0 otherwise
$C_{i}^{min}$	Minimum number of care workers with QL i or higher
${\overset{ˉ}{C}}_{i}$	Maximum number of care worker hours of QL i available
$L_{it}$	Number of resident requiring activity of QL i at time t
Decision variables	
$x_{ik}$	Number of care workers with QL i for shift type k
$c_{it}$	Staffing level of care workers with QL i during time interval t
$c_{it}^{*}$	Number of care workers working at QL i during time interval t
$q_{it}$	Backlog of work requiring QL i at the start of time interval t

The objective is to minimise the total backlog (1). Equation (2) gives the available capacity of QL i at time t in terms of the shifts. Equation (3) guarantees that the available hours are not exceeded. Equations (4) and (5) model the hierarchical qualification levels. The first equation (4) dictates that the actual number of care workers performing a level i activity should not exceed the total number of care workers that are qualified to perform that activity. The second equation (5) makes sure that the care workers are not simultaneously used for more than one QL. When there are only two QLs (say i and i+1), the two constraints can be simplified to

$$c_{it}^{*}\leq \;c_{it}+c_{i+1,t},\;\;c_{i+1,t}^{*}\;\leq \;c_{i+1,t},\;\;\;\;\;\;\;\;\;\;\;\forall t\in T$$

$$c_{it}^{*}+c_{i+1,t}^{*}\leq \;c_{it}+c_{i+1,t},\;(c_{i+1,t}^{*}\leq c_{i+1,t}),\;\forall t\in T$$

The recursive relation for the backlog of level i activities is given in Equation (6), whereas Equation (7) provides that there is no backlog at the end of the day. Observe that the problem becomes infeasible if the number of available hours is not sufficient. The backlog at the end of the day may also be transformed into a soft constraint by including it in the objective function, but we find that such a situation indicates that there is a structural problem and prefer a hard constraint. Equation (8) ensures that the minimum number of required care workers are present. Equation (9) provides that backlogs are non-negative, whereas Equation (10) and Equation (11) make sure that staffing levels and number of shifts scheduled are integer values.

### 4.3. Step 3: task assignment

We now turn to step 3 of the conceptual framework of Figure 1. This step is at the operational level rather than at the tactical level, but it is required to determine the performance of a capacity plan in terms of waiting time and overtime.

In the current practice, usually no sophisticated algorithms are applied to assign tasks to workers. Hence, tasks are often assigned in an ad hoc manner, which is very similar to a First Come First Served approach (FCFS). In this paper, a genetic algorithm (GA) is developed that solves the task assignment in a more sophisticated way (see, Subsection 4.3.2). A greedy heuristic, which resembles the current FCFS assignment approach, is also developed in order to compare the performance of the current practice to the performance of the GA (see, Subsection 4.3.1).

To describe the two task assignment algorithms, we need to define some notation first. The shifts are determined from the shift scheduling algorithm presented in the previous section (step 2 of Figure 1). Denote by J the total number of shifts during the time horizon. Let $S_{j}$ and $E_{j}>S_{j}$ be the start and end times of shift j∈{1,…,J}, respectively, as determined by the shift scheduling algorithm in step 2. For convenience, we number the shifts in decreasing QL. Let $s_{i}$ be the number of shifts that can handle activities of the i th largest QL. Then, an activity with QL i can be handled by shifts $1,\ldots ,s_{i}$.

#### 4.3.1. Greedy heuristic

In the greedy heuristic, we assign activities directly to the shift that becomes available first. We present a recursive scheme that can be used to determine waiting times and assignment of tasks. The recursion is essentially based on the Kiefer-Wolfowitz recursion for the G/G/s queue. The difference with the G/G/s queue is that we need a multi-class system and that the number of servers vary over time (but we are primarily interested in the deterministic version).

It is convenient to order the activities according to their preferred starting time (in increasing order). Below, when we refer to activity n, we understand this activity to be ordered according to the PAT. Denote by $A_{n}=T_{n+1}-T_{n}$ the interarrival time between activities n and n+1 (possibly being equal to 0).

Let $V_{t}^{j}$ be the remaining time until shift j∈{1,…,J} becomes available at time t. We extend $V_{t}^{j}$ to the negative half line and let $V_{t}^{j}<0$ denote that shift j is already idle for $|V_{t}^{j}|$ time units. Note that $V_{t}^{j}$ decreases linearly in t and makes a jump when an activity is assigned to shift j. We are particularly interested in $V_{t}$ just before PAT instants, i.e., the n th activity observes $W_{n}=(W_{n}^{1},\cdots ,W_{n}^{J})=V_{{T_{n}}^{-}}$, where $V_{t^{-}}=\lim_{s↑t}V_{s}$. Also, let ${\hat{W}}_{n}$ denote the availability of shifts just after activity n has been assigned.

Now, we start the recursion at time 0 with ${\hat{W}}_{0}^{j}=S^{j}$, for j=1,…,J, as shift j will be available at its starting time. Next, use the recursive relation

$$W_{n}^{j}={\hat{W}}_{n-1}^{j}-A_{n-1}.$$

Observe that shift j may be working in overtime after the n th activity in case $T_{n}+(W_{n}^{j}{)}^{+}>E^{i}$, where $x^{+}=\max(x,0)$. In particular, the overtime is given by ${\left(T_{n}+{\left(W_{n}^{j}\right)}^{+}-E^{j}\right)}^{+}$. Now, an activity will be assigned to a compatible shift resulting in the smallest waiting time for this activity. If all shifts have finished, e.g., at the end of the day, then the activity will be assigned to a compatible shift resulting in the smallest overtime. Specifically, activity n will be assigned to shift

$$s_{n}^{*}=\arg\min\{j\leq s_{QL_{n}}:W_{n}^{j}+M{\left(T_{n}+{\left(W_{n}^{j}\right)}^{+}-E^{j}\right)}^{+}\},$$

with M a sufficiently large number. Consequently, just after the assignment, we have

$${\hat{W}}_{n}^{j}=\left\{\begin{array}{cc} W_{n}^{j}, & if\;\;j\neq s_{n}^{*},\\ {\left(W_{n}^{j}\right)}^{+}+B_{n}, & otherwise\;\; \end{array}\right.$$

The waiting time of activity n is now $(W_{n}^{s_{n}^{*}}{)}^{+}$.

#### 4.3.2. Genetic algorithm

We now present a more advanced algorithm to allocate care workers to activities and to determine the moment at which activities should be carried out. The objective is to create a feasible task schedule that minimises a weighted combination of the total earliness and waiting time for nursing home residents.

In order to solve the task scheduling problem at-hand, we iteratively carry out a two-step procedure. In each generation of the GA, we first determine which care worker should perform which task in which order. Next, we determine the optimal starting times for the care tasks. For this second step, we use an efficient LP approach, which is not straightforward to incorporate in an GA. To the best of the authors’ knowledge, this the first study in which such an approach is applied. These two steps together yield the fitness of a candidate solution and are used in the GA to find better solutions.

**Schedule representation** A task schedule is described by a vector representation $\left(a_{1},\ldots ,a_{N}\right)$, where $a_{n}\in \{1,\ldots ,J\}$ is the care worker that carries out activity n. For instance, with N=6 and J=3, the solution (2,3,1,2,2,1) denotes that activities 3 and 6 are carried out by care worker 1, activities 1, 4, and 5 are done by care worker 2 and care worker 3 only takes care of activity 2. During a single shift, activities are carried out in the order of the individual PATs.

**Outline GA procedure** The outline of the GA is based on Eiben & Smith (2015), Chapter 3, and is presented in Algorithm 1. A population is a collection of schedules. In each iteration of the GA, a collection of children is created by using “crossover” and “mutation” operations on parent solutions. Subsequently, the new generations should consist of better schedules than the old generations. We discuss steps 2–5 of the algorithm in more detail below.

**Table:** 

**Algorithm 1** Outline GA procedure
**initialize** population with random candidate solutions
**repeat**
**1. Select**parents
**2. Crossover** pairs of parents
**3. Mutate** resulting offspring
**4. Evaluate** new candidates ▷ incl. start time of activities
**5. Select** individuals for next generation **until** time limit exceeds or no improvements for x generations

**Initialise** First, we create a (random) population of initial solutions. This is done by randomly assigning the activities to care workers who work during the preferred time of the activities. We also allow that activities are carried out by care workers that do not work at the preferred time of the activity. An activity is assigned to a care worker who does not necessarily work during the preferred time with a probability of 0.1.

**Crossover** For a combination of parents, a crossover point P is generated at random. A child is created by selecting elements 1 to P from the mother and elements P+1 to N from the father. The second child inherits elements 1 to P from the father and elements P+1 to N from the mother. The crossover operation is performed on two parts of the population. First of all, the crossover operation is performed on the best $G_{C}$% of the population. The best solution of the population is combined with the second-best solution; the third-best solution with the fourth-best solution, etcetera. In addition, the crossover operation is performed on combinations of randomly chosen solutions. Each solution can only be chosen once.

**Mutate** Each solution is mutated. With this mutation operator, each activity is assigned to another randomly selected care worker with probability $p_{m}$. Thus, the value of $p_{m}$ determines to which extent solutions are mutated; larger values of $p_{m}$ imply more mutations. Then, with probability $p_{s}$, the activity is allocated to a care worker that works during the preferred time and has a compatible QL. Here, a compatible QL means that it is equal or larger than the QL required for the activity. Otherwise, a care worker is selected that does not necessarily work during the preferred time while still having a compatible QL.

The mutation operation described above may lead to quite disruptive changes. This is highly valuable for maintaining diversity in the generation, but makes it more difficult to create similar solutions. In the final phase of the search process, smaller adaptations are useful for fine-tuning of the good individuals. Therefore, a second type of mutation is performed on $G_{FM}$% of the population in each generation. This type of mutation is specifically designed to mutate the first activity in the schedule for which waiting occurs, say activity i, and the neighbouring activities. We take a number $A_{FM}$ randomly between 1 and 4. The $A_{FM}$ activities directly before i and the $A_{FM}$ activities directly after i are mutated in a similar way as described above. That is, each of these activities are assigned to another randomly selected care worked with probability $p_{FM}$. With probability $p_{s}$, the activity is allocated to a care worker that works during the preferred time and has a compatible QL. Otherwise, a care worker is selected that does not necessarily work during the preferred time while still having a compatible QL.

**Evaluate** In the evaluation step, the fitness is calculated of all newly created solutions. The fitness consists of two elements: (i) total waiting time and earliness corresponding to PATs, and (ii) total overtime of care workers. Note that the schedule representation determines which activity is carried out by which care worker and in which order, but not the time at which the activity should be performed. The time at which activities are performed are, however, required to determine the quality of the schedule. We employ an LP model to determine these activity times.

In the following, we introduce the LP model that is applied to determine the starting time of all activities. Let $N_{j}$ be the number of activities that is carried out by care worker j∈{1,…,J}, and we drop the shift number from the notation for now. During the shift, the objective is to minimise the weighted sum of earliness and tardiness. Let $C_{W}$ and $C_{E}$ denote the corresponding weights. Define the decision variables $x_{n}$ as the starting time of the n-th activity, $n=1,\ldots ,N_{j}$. Observe that the waiting time of the n-th activity is $\max\{x_{n}-T_{n},0\}$. We use the auxiliary variables $y_{n}$, $n=1,\ldots ,N_{j}$, to linearise the model. Similarly, the earliness of the n-th activity is $\max\{T_{n}-x_{n},0\}$, for which we use the auxiliary variables $y_{n}$ for $n=N_{j}+1,\ldots ,2N_{j}$. This gives rise to the following simple LP:

(12) $$Minimise\;\;\;C_{W}\sum_{n=1}^{N_{j}}y_{n}+C_{E}\sum_{n=N_{j}+1}^{2N_{j}}y_{n}$$

(13) $$subject\;\;to\;\;\;x_{1}\geq S_{j},\;\;x_{N_{j}}\leq E_{j}$$

(14) $$x_{n}+B_{n}\leq x_{n+1},\;\;\;\;\;\;\;\;\;\;\;\;\;\;\;\;\;\;\;\;\;\;\;\;\;\;\;\;n=1,\ldots ,N_{j}$$

(15) $$y_{n}\geq x_{n}-T_{n},\;\;\;\;\;\;\;\;\;\;\;\;\;\;\;\;\;\;\;\;\;\;\;\;\;\;\;\;\;\;\;n=1,\ldots ,N_{j}$$

(16) $$y_{N_{j}+n}\geq T_{n}-x_{n},\;\;\;\;\;\;\;\;\;\;\;\;\;\;\;\;\;\;\;\;\;\;\;\;\;n=1,\ldots ,N_{j}$$

(17) $$x_{n},y_{n},y_{N_{j}+n}\geq 0,\;\;\;\;\;\;\;\;\;\;\;\;\;\;\;\;\;\;\;\;\;\;\;n=1,\ldots ,N_{j}$$

Note that the LP has to be solved quite often, i.e., for each shift and each individual. However, solving this LP is very fast. Moreover, once the LP has been solved, its solution is saved, so that it can be re-used in a later generation if the same LP has to be solved again.

**Select** A new generation is selected by roulette wheel selection from the previous generation and the newly generated offspring population. Solutions are selected based on their fitness. A higher fitness corresponds to a higher probability of being part of the next generation.

**Parameter tuning** An important element of an GA is the tuning of its parameters. In the developed algorithm, five parameters should be set: $G_{C}$, $G_{FM}$, $p_{FM}$, $p_{m}$ and $p_{s}$. Tuning the parameters of an GA is a cumbersome process, as the combination of parameters influences the performance of the GA. As it is computationally impossible to consider all parameter combinations (for each parameter combination the GA would have to be run multiple times), we use a search algorithm, namely Hyperopt (Bergstra et al., 2013). This algorithm, specifically developed for hyperparameter tuning, searches the total parameter space in a sophisticated way. We use the TPE algorithm, as the fitness evaluations are computationally costly and thus a small evaluation budget is available. We refer the interested reader to Bergstra et al. (2011) for details about the TPE algorithm.

## 5. Numerical experiments

The aim of this section is to provide insight in the potential benefits of using the capacity planning process as described in Section 4. To do so, we compare the performance of three different planning strategies under different scenarios. The main characteristics of the applied strategies are presented in Table 4. Strategies A and B make use of the shift scheduling approach as presented in Section 4.2. Strategy C, on the other hand, resembles current practice. In order to create shift schedules that resemble current practice as closely as possible, we used worker-to-resident ratios that are based on a study of the Dutch Institute for Health Services Research (NIVEL), see, Hingstman et al. (2012) or Bekker et al. (2019). Specifically, we used an ILP in the spirit of Section 4.2 to choose shifts such that the worker-to-resident ratios across the day, as published by NIVEL, are matched as closely as possible. For the assignment of tasks, strategy A makes use of the GA that is presented in Section 4.3.2, whereas strategies B and C use the greedy heuristic (see, Section 4.3.1). The greedy heuristic closely resembles the way in which tasks are assigned in the current practice.

**Table 4.** Overview of the applied planning strategies.

	Planning strategy		
	A	B	C
Shift scheduling	MILP	MILP	NIVEL
Task assignment	GA	Greedy	Greedy

Regarding the numerical experiments, it should be noted that the GA involves randomness, that is, the solutions will differ each time the algorithm is run. Therefore, for each experiment, the algorithm is run 100 times to obtain insight in the variability of the fitness.

### 5.1. Scenarios & base case

For the purpose of this numerical experiment, the performance of the three planning strategies has been compared under different scenarios. Regarding the scenarios, a change in the following aspects have been taken into account: (1) the number of clients (i.e., the effect of less or more clients), (2) qualification levels (i.e., the effect of working with or without QLs), and (3) utilisation of care workers (i.e., the effect of a higher average workload per care worker). Table 5 provides a detailed overview of how the changes in the aforementioned aspects have been operationalised into (sub-)scenarios. The current practice situation (see, Section 3) is represented by the following sub-scenario: *Base case QL U*. In other words, a situation with 18 clients in care (i.e., base case), where QLs are used and in which the average utilisation is 70%. Some additional characteristics of the current situation are:

**Table 5.** Overview of aspects examined in the scenario analysis, with in grey the characteristics of the current practice situation.

Aspect	Description	Referred to as
Number of clients	18	Base case
	14	Scenario 1
	22	Scenario 2
Qualification Levels	With	QL
	Without	no QL
Utilisation	70%	U
	74%	U+
	84%	U++

• Number of tasks per day: 53 and 52 for QL2 and QL3.

• Available capacity per day: 18 h for both QL2 and QL3.

• Available shift lengths: 4, 6, and 8 h.

• Utilisation per QL: 81% and 59% for QL2 and QL3.

An overview of the main characteristics of the U QL-version of the base case, scenario 1 and scenario 2 can be found in Table 6.

**Table 6.** Main characteristics of the U QL-version of the base case, scenario 1 and scenario 2.

	# Tasks		Capacity		Shifts lengths
	QL2	QL3	QL2	QL3	
Base case	53	52	18 h	18 h	4,6, and 8 h hrs
Scenario 1	38	42	14 h	14 h	
Scenario 2	68	62	22 h	22 h	

### 5.2. Running time & parameter tuning

In terms of computational time, we note that the computation times for planning strategies B and C (see, Table 4) are negligible; in some cases, the MILP (i.e., the shift scheduling of step 2) takes several seconds. Planning strategy A requires more computational budget, due to the (more advanced) GA used for assigning the tasks in step 3. One run of the GA takes between 1.92 and 14.49 minutes with a population of 200 schedules and 100 generations, depending on the experiment.^1^

**Figure 4.**
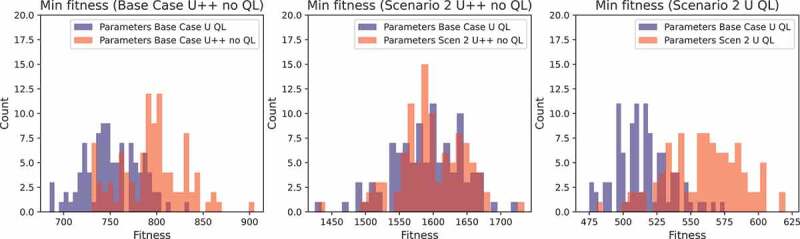
The minimum fitness for three scenarios and two parameter settings.

The computation time of the parameter optimisation of the GA is considerable (around 24 h) and therefore it is preferred to only perform this optimisation once (i.e., not for each new sub-scenario again). Extensive testing indicates that running the parameter optimisation on sub-scenario *Base case U QL* creates an algorithm that is sufficiently versatile and yields good results on the other sub-scenarios. This is shown for three different sub-scenarios in Figure 4. To create these figures, the GA was run 100 times on a certain sub-scenario with two parameter settings: the first parameter setting obtained from a parameter optimisation on the Base Case U QL sub-scenario and the second parameter setting obtained from a parameter optimisation on the specific sub-scenario. Clearly, the results obtained by running the GA with the Base Case U QL sub-scenario parameters results in equal or even better fitness values than the results obtained with the parameters for each specific sub-scenario. Thus, by using the parameters of the Base Case U QL sub-scenario, an algorithm is created that can be applied in diverse settings while keeping the running time and results acceptable, as only one parameter optimisation has to be done in total, instead of one per sub-scenario. The output of the parameter optimisation for the Base Case U QL scenario can be found in Table 7. These parameters have been used to generate the results in the following subsections. Interestingly, both the values for $G_{S}$ and $p_{s}$ are high, which means that a large percentage of the best population is used in the crossover population and that an activity has a large probability to be allocated to a care worker that works during the preferred time and has a compatible QL.

**Table 7.** The used parameters found by running the parameter optimisation on base case U QL.

Parameter	Value
$G_{C}$	0.631
$G_{FM}$	0.080
$p_{s}$	0.929
$p_{m}$	0.050
$p_{FM}$	0.089

### 5.3. Experiments with QLs

Regarding the results of the numerical experiments, we distinguish between working with QL (i.e., this section) and working without QLs (Section 5.4). Furthermore, the base case (see, Section 5.1) will serve as our primary example. As previously discussed, three experiments with different utilisation levels are considered (“U”, “U+”, and “U++”). The performance of each of the presented planning strategy is being discussed following the three steps as presented in Section 4.

#### 5.3.1. Planning strategy A

**Step 1**: We refer to Figure 3 for the evolution of the workload across the day. There is a considerable amount of variability in the workload across the day, which corresponds to the natural rhythm in daily living. Note that step 1 is equal for all three utilisation levels.

**Step 2**: The shifts that are obtained using the shift scheduling algorithm can be found in Table 8. Observe that most of the capacity is used in the morning, which is also in line with the workload across the day that follows from step 1 (Figure 3). Observe that in case of the “U+” and “U++” scenarios, no care workers are scheduled during the afternoon (12:30–15:00, 13:30–16:30, respectively). This follows from the fact that the amount of overcapacity is small (high utilisation) and there are only a few healthcare tasks during this time interval. Hence, care workers can then be used more efficiently during the busier hours. The shifts for scenarios 1 and 2 can be found in the appendix (see Table A1 and A3).

**Table 8.** Shifts for base case experiments with QLs (Step 2).

Scenario	Number of shifts	Qualification Level	START	END	Utilisation
Base case U QL	2	2	07:00	13:00	QL2 81%
	1	3	07:30	11:30	QL3 59%
	1	3	08:30	12:30	Total 70%
	1	2	11:00	17:00	
	1	3	16:00	22:00	
	1	3	19:00	23:00	
Base case U+ QL	2	2	07:30	13:30	QL2 81%
	1	3	07:30	11:30	QL3 67%
	1	3	08:30	12:30	Total 74%
	1	3	15:00	23:00	
	1	2	16:30	22:30	
Base case U++QL	2	1	07:30	13:30	QL2 81%
	1	3	07:30	13:30	QL3 89%
	1	2	16:30	22:30	Total 84%
	1	3	17:00	23:00	

**Figure 5.**
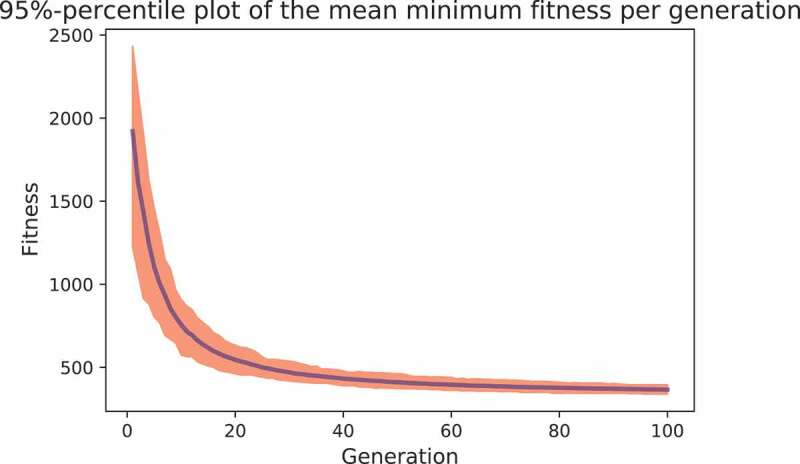
The mean minimum fitness per generation together with a 95% empirical confidence interval.

**Step 3**: In this step, we need to assign tasks to care workers, from which we may determine the waiting time of clients and possible tardiness of care workers. We do so by using the developed GA. As previously indicated, the population size is set to 200, and the number of generations is set to 100. We report the fitness of the best individual of the final generation, hereafter referred to as the minimum fitness. As a GA is a randomised algorithm, we run the GA 100 times. Figure 5 presents the mean minimum fitness per generation together with a 95% confidence interval. Clearly, the strongest reduction in the minimum fitness is obtained in the first few generations. Table 9 reports the mean (minimum) fitness together with its standard deviation.

**Table 9.** Results for strategy A, *with* QLs, based on 100 runs.

Experiment		Min fitness
		μ±σ
Base case	U QL	345.50 ± 10.43
	U+ QL	570.65 ± 25.33
	U++ QL	2020.45 ± 25.22
Scenario 1	U QL	415.45 ± 1.43
	U+ QL	768.55 ± 6.61
	U++ QL	827.00 ± 5.61
Scenario 2	U QL	514.00 ± 19.79
	U+ QL	1060.15 ± 24.41
	U++ QL	3450.15 ± 78.56

An utilisation of 70% (over the two QL’s) leads to acceptable waiting times of a few minutes on average. For instance, in the base case, the total waiting time is 345.5 minutes over 105 activities yielding 3.29 minutes on average. Observe that the fitness strongly increases with utilisation. For the base case and utilisations over 80% (scenario “U++”), the overtime amounts to 210 minutes in total and excessive waiting occurs (approximately 1810 minutes in total, yielding a fitness of about 2020). Hence, due to the variability in healthcare tasks across the day, it seems difficult to operate at high utilisations. This is recognised in practice, where nursing homes work with an utilisation of about 70%.

Furthermore, from the experiments, we see some differences between the fitness of the 200 individuals of the final generation (not reported here for conciseness). This shows that there is some diversity within the final generation. From the standard deviations σ in Table 9, we see a quite stable performance of the best individual.

#### 5.3.2. Planning strategies B and C

We now compare the results of planning strategy A with strategies B and C. As there is no structural monitoring of the workload across the day, step 1 does typically not occur in the current situation (i.e., planning strategy C).

**Step 2**: For the planning strategy B, we use the MILP of Subsection 4.2 resulting in the same shifts as in Table 8. Regarding strategy C, the aim was to determine shifts such that the current worker-to-resident ratios across the day are maintained as closely as possible. We refer to Hingstman et al. (2012) and Bekker et al. (2019) for these ratios that tend to lead to a rather stable capacity across the day, with a small peak during the morning. As the workload deviates from this pattern, there seems to be much room for improvement here.

**Step 3**: For both strategies B and C, the greedy algorithm of Subsection 4.3.1 is used to assign tasks to care workers. We determined the total waiting and overtime for both strategies, yielding the corresponding fitness. Moreover, to compare the performance with strategy A, we define the relative difference for strategy i=B,C with strategy A as

$$\Delta_{min}=\frac{f_{i}-f_{A}}{f_{A}}\times 100\%,$$

where $f_{j}$ denotes the (mean) fitness of strategy j=A,B,C. The results can be found in Table 10. Clearly, an enormous gain in performance can be achieved by determining shifts of care workers (step 2). The fitness values of strategy C are multiple hundreds of percents off from the fitness of strategy A; for instance, for the base case under sub-scenario “U” the performance of strategy C is 748% worse than strategy A. This reveals the huge efficiency potential in nursing home care. Strategy B clearly outperforms strategy C, revealing the importance of a good shift schedule. However, using the GA (i.e., strategy A) still reduces the fitness by tens of percents compared to strategy B. Hence, in the base case under sub-scenario “U” strategy B still performs 23% worse than strategy A. In particular, if the utilisation becomes higher (scenario “U++”), the benefits of the GA become larger. This may be explained by the fact that the impact of scheduling decisions becomes larger due to the tight capacity.

**Table 10.** Benchmark results, with QL.

		Fitness and % improvement			
Experiment		Strategy B	$\Delta_{min}$	Strategy C	$\Delta_{min}$
Base case	U QL	425.00	23%	2930.00	748%
	U+ QL	980.00	72%	5267.00	823%
	U++QL	3190.00	1347%	10,395.00	4615%
Scenario 1	U QL	655.00	58%	2415.00	481%
	U+ QL	1670.00	117%	3003.00	291%
	U++QL	2710.00	228%	8435.00	920%
Scenario 2	U QL	740.00	44%	3515.00	584%
	U+ QL	1465.00	38%	4470.00	322%
	U++QL	7525.00	118%	7345.00	113%

### 5.4. Experiments without QLs

For the experiments presented in this subsection, we neglect QLs and assume that every care worker is qualified to handle every activity, that is, every care worker is assumed to have the highest QL. Again, the base case will serve as our primary example, and we consider as in 5.3 three levels of utilisation.

#### 5.4.1. Planning strategy A

**Step 1**: The workload is now just the sum of the workloads of the two QLs as displayed in Figure 3, and again equal for all utilisation levels. Note that the workload during the morning and evening becomes more stable during these periods by neglecting QLs. But still, there is considerable variability across the day.

**Step 2**: The results of the shift scheduling algorithm can be found in Table 11. As expected, most of the capacity is assigned to the morning again in all experiments, with 3–4 out of 6 care workers between 11:00 and 13:00 hours. However, compared to the situation with QLs, a small part of the capacity is shifted from the morning to the afternoon. When combining the work of the QLs, it can be seen from Figure 3 that 3 care workers during 7:30–11:00 hours are tight, but are just enough to handle the aggregated demand. The shifts for scenarios 1 and 2 can be found in the appendix (see Table A2 and A4).

**Step 3**: The GA is again used to assign activities to care workers in this step. The mean minimum over 100 evaluations can be found in Table 12, together with the standard deviations. Table 12 is similar to Table 9, but without the distinction in QLs. Comparing the two tables, it evidently holds that discarding QLs substantially improves performance. Moreover, we see that for higher utilisations, the reduction in fitness value is typically larger when discarding QLs, for example, for the base case the reductions are about 18%, 42%, and 63% for scenarios “U”, “U+”, and “U++”, respectively. This can be explained by the fact that discarding QLs provides more flexibility in task assignment, which may be more beneficial in scenarios with high utilisations. We like to note that the precise percentages clearly depend on the situation, with the workload pattern as a predominant factor. Finally, as we also concluded with QLs, we see a quite stable performance of the minimum fitness of the GA again, as can be concluded from the relatively small standard deviation σ.

#### 5.4.2. Planning strategies B and C

Again, we compare the results of planning strategy A with strategies B and C to gain insight into the impact of QLs.

**Step 2**: We determined the shifts of care workers using the same methods as for the case with QLs. That is, for strategy B the shifts are based on the MILP after incorporating the aggregated workload across the day, yielding Table 11. The shifts for planning strategy C are based on the same worker-to-resident ratios again. As expected, regarding strategy C the shifts do not well align with the workload pattern.

**Step 3**: We compare the fitness of the two benchmark strategies B and C in Table 13 with strategy A; $\Delta_{min}$ again shows the relative difference in fitness between the benchmark strategies B and C, and strategy A. The difference is enormous again, where clearly huge gains can be achieved by choosing shifts in a more appropriate manner. Comparing the $\Delta_{min}$ of strategy B with that of A, we still see significant gains in performance ranging from 9% to 45%. However, the added value of the GA is not as large as in the case with QL. This can be explained from the observation that without QLs, simple heuristic rules work better. For instance, there is no need for care workers of QL3 to make a trade-off whether they should now carry out a type-2 activity or remain idle for a future type-3 activity.

**Table 11.** Shifts per scenario (step 2), without QLs.

Scenario	Number of shifts	Qualification Level	START	END	Utilisation
Base case U no QL	3	3	07:00	13:00	70%
	1	3	11:00	17:00	
	1	3	15:00	23:00	
	1	3	18:30	22:30	
Base case U+ no QL	3	3	07:00	13:00	74%
	1	3	11:00	17:00	
	1	3	16:00	22:00	
	1	3	19:00	23:00	
Base case U++ no QL	3	3	07:30	13:30	84%
	1	3	14:30	22:30	
	1	3	18:30	22:30

**Table 12.** Results for strategy A, *without* QLs, based on 100 runs.

Experiment		Min fitness
		μ±σ
Base case	U no QL	283.60 ± 17.41
	U+ no QL	329.30 ± 18.28
	U++ no QL	753.10 ± 29.51
Scenario 1	U no QL	207.15 ± 9.33
	U+ no QL	373.95 ± 13.33
	U++ no QL	878.15 ± 35.00
Scenario 2	U no QL	406.45 ± 18.63
	U+ no QL	682.20 ± 30.38
	U++ no QL	1591.10 ± 51.53

**Table 13.** Benchmark results, without QL.

		Fitness and % improvement			
Experiment		Strategy B	$\Delta_{min}$	Strategy C	$\Delta_{min}$
Base case	U no QL	335.00	18%	1460.00	415%
	U+ no QL	410.00	24%	1975.00	500%
	U++ no QL	1040.00	38%	4680.00	521%
Scenario 1	U no QL	225.00	9%	1250.00	503%
	U+ no QL	445.00	19%	1985.00	431%
	U++ no QL	1270.00	45%	2775.00	216%
Scenario 2	U no QL	455.00	12%	2430.00	498%
	U+ no QL	840.00	23%	3205.00	370%
	U++ no QL	2195.00	38%	5635.00	254%

## 6. Conclusions & discussion

We presented a three-step conceptual framework that can be used to structure the care-related capacity planning process in a nursing home context. More specifically, we identified the following steps: (1) workload evaluation, (2a) staffing, (2b) shift scheduling, and (3) rostering & tasks assignment. For step 2b (shift scheduling) we presented an MILP, which can be used in a setting with hierarchical qualifications. In addition, for the task assignment in step 3 we proposed a modified genetic algorithm, which determines optimal starting times of activities using an LP next to the assignment of activities to care workers. By benchmarking the proposed framework against the current situation, it is shown that enormous improvements can be achieved in terms of efficiency and waiting time. Specifically, it can be observed that appropriate shift scheduling is crucial to match the available capacity with demand. However, using a generic algorithm for task assignment also provides considerable improvements. The numerical experiments show that applying the proposed framework results in an average waiting time of only a few minutes for an average occupancy of near 70%, despite the considerable variability in demand across the day. For utilisations of over 80%, the waiting and overtime seem to increase rapidly. As such, this study reveals the potential of efficiently organising client-centred care using an appropriate optimisation framework.

The proposed framework provides a sound practical vehicle to organise client-centred care without overstretching available capacity. From a practical point of view, we see that it is difficult for nursing homes to give preferences of clients a more prominent place. This requires registration of client preferences, which is not yet common. Also, we like to stress that the shift schedule and activity planning provides a blueprint for practice. Events happening during the day may require care workers to deviate from the activity plan.

An appealing feature of the three-step framework is that the capacity-planning process can be divided into separate steps. These separate steps provide more insight in the process and will be easier to implement in practice. For instance, in view of the autonomy of the care worker, we envisage that in practice it will be simpler to modify the shifts schedule than the task assignment. Also, the decomposition better allows for modifications in the separate steps. The take assignment results with a genetic algorithm are very promising. An interesting topic for further research is to compare the performance of the GA algorithm with other optimisation procedures. Moreover, the results can be improved by integrating steps 2 and 3, but this will make the optimisation model more complex and the advantage of decomposing the capacity planning process will be lost. Finally, it is of interest to apply this framework to situations where intra- and extramural care are combined.

## Note

1. Experiments have been carried out on an 2.7 GHz Quad-Core Intel Core i5 with 16GB of RAM.

## Appendix A. Shifts scenarios 1 and 2

**Table A1.** Shifts for each experiment for scenario 1, with QLs.

Scenario	Number of shifts	Qualification Level	START	END	Utilisation
Scenario 1 U QL	1	2	7:30	11:30	QL2 81%
	1	2	7:30	13:30	QL3 59%
	1	3	7:00	13:00	Total 70%
	1	3	15:00	23:00	
	1	2	18:30	22:30	
Scenario 1 U+ QL	1	2	7:30	11:30	QL2 85%
	1	2	9:00	13:00	QL3 69%
	1	3	7:30	13:30	Total 77%
	1	3	17:00	23:00	
	1	2	18:00	22:00	
Scenario 1 U++ QL	1	2	7:30	11:30	QL2 85%
	1	3	7:30	13:30	QL3 83%
	1	2	9:00	13:00	Total 84%
	1	2	18:00	22:00	
	1	3	19:00	23:00	

## Appendix

**Table A2.** Shifts for each experiment for scenario 1, without QLs.

Scenario	Number of shifts	Qualification Level	START	END	Utilisation
Scenario 1 U no QL	2	3	7:00	13:00	70%
	1	3	8:00	12:00	
	1	3	16:00	22:00	
	1	3	17:00	23:00	
Scenario 1 U+ no QL	1	3	7:30	11:30	77%
	1	3	7:30	13:30	
	1	3	8:30	12:30	
	1	3	16:00	22:00	
	1	3	19:00	23:00	
Scenario 1 U++ no QL	1	3	7:30	11:30	84%
	1	3	8:00	12:00	
	1	3	10:00	14:00	
	1	3	16:00	22:00	
	1	3	19:00	23:00	

## Appendix

**Table A3.** Shifts for each experiment for scenario 2, with QLs.

Scenario	Number of shifts	Qualification Level	START	END	Utilisation
Scenario 2 U QL	2	2	7:00	13:00	QL2 78%
	2	3	7:00	13:00	QL3 58%
	1	2	11:00	17:00	Total 68%
	1	3	16:00	22:00	
	1	2	18:30	22:30	
	1	3	19:00	23:00	
Scenario 2 U+ QL	2	2	7:00	13:00	QL2 78%
	1	3	7:30	11:30	QL3 71%
	1	3	9:00	13:00	Total 75%
	1	2	11:00	17:00	
	1	3	16:00	22:00	
	1	2	18:30	22:30	
	1	3	19:00	23:00	
Scenario 2 U++ QL	1	2	7:30	11:30	QL2 86%
	1	2	7:30	13:30	QL3 92%
	1	3	7:30	13:30	Total 88%
	1	2	11:00	17:00	
	1	3	18:00	22:00	
	1	2	18:30	22:30	
	1	3	19:00	23:00	

## Appendix

**Table A4.** Shifts for each experiment for scenario 2, without QLs.

Scenario	Number of shifts	Qualification Level	START	END	Utilisation
Scenario 2 U no QL	4	3	7:00	13:00	68%
	1	3	11:00	17:00	
	1	3	16:00	22:00	
	1	3	18:30	22:30	
	1	3	19:00	23:00	
Scenario 2 U+ no QL	1	3	7:30	11:30	75%
	2	3	7:30	13:30	
	1	3	8:00	12:00	
	1	3	11:00	17:00	
	1	3	16:00	22:00	
	1	3	18:30	22:30	
	1	3	19:00	23:00	
Scenario 2 U++ no QL	2	3	7:30	13:30	88%
	1	3	8:00	12:00	
	1	3	8:30	12:30	
	1	3	15:30	21:30	
	2	3	19:00	23:00	
